# Simulation and comparison of objectively graded microsurgery steps by using a microscope and Symani robotic system by young professionals

**DOI:** 10.1007/s11701-025-02490-3

**Published:** 2025-06-17

**Authors:** Henning Wieker, Niklas Brandenburg, Dorothee Spille, Juliane Wagner, Jan-Tobias Weitkamp, Jörg Wiltfang, Johannes Spille

**Affiliations:** 1 Department of Oral and Maxillofacial Surgery, Christian Albrechts University, University Hospital of Schleswig-Holstein, Campus Kiel, Arnold-Heller-Straße 3, 24105 Kiel, Germany; 2 Department of Neurosurgery, University Hospital Münster, Münster, Germany

**Keywords:** New technology, Students, Surgical education, Anastomosis, Hand–eye coordination

## Abstract

The aim of the current study was to compare the accuracy of objectively graded microsurgery steps by young professionals using a microscope and a microsurgery robot. 40 students performed three exercises with a dental surgical operating microscope and a microsurgery robot. The exercises consisted of grasping and placing a needle, threading the needle, and performing a surgical knot. All students successfully completed the exercises with the microscope and the Symani. The microscope demonstrated significantly shorter operating times for grasping and placing a needle (*p* = 0.003) and for a single-button suture of an anastomosis (*p* < 0.001). Gaming, sewing, or knitting had a significant advantage for both the microscope and the Symani for grasping and placing a needle (*p* < 0.001). Young professionals appear to be able to work adequately with both the microscope and the robot in the surgical model for anastomoses. The students demonstrated a strong interest in adopting robotic technologies, which is why implementing robotics in the clinics' teaching process is necessary. This could inspire the next generation of medical professionals for robotic surgery and microsurgery, as well as support their career path.

## Introduction

In recent decades, microsurgery has developed steadily due to technical innovations, including the use of microscopes. In this way, microvascular surgery and microneurosurgery have shown revolutionary advances in clinical replantation and transplantation of composite tissues, as well as other allotransplantations [1]. In oral and maxillofacial surgery, free grafts and microsurgical anastomoses enable the reconstruction of larger facial and oral region defects. The complication rate is described in the literature as low, although some complications and graft losses do occur with microsurgical anastomosis [2, 3]. All operations aim to reduce invasiveness and complication rates while increasing effectiveness. Robotic surgery, especially the Da Vinci Surgical System, has revolutionized numerous surgical specialties, including urology, gynecology, general surgery, otolaryngology, cardiothoracic surgery, and neurosurgery, enabling surgeons to perform complex procedures with greater precision and accuracy [4]. Moreover, the Da Vinci surgical system is continually being further developed to expand its surgical capabilities [5]. However, mastering minimally invasive robotic surgery is technically demanding and requires intensive and continuous training [6].

Especially for microsurgery and supermicrosurgery, the Symani Surgical System^®^ (Medical Microinstruments, MMI, Calci, Italy) was developed, which enables plastic reconstruction with vascular and nerve as well as lymph node anastomoses [7]. The Symani has two robotic arms, which have the world’s smallest surgical instruments and are designed to extend a surgeon’s range of movement beyond the capabilities of the human hand [8]. Previous studies have shown that the Symani can utilize its advantages, as reducing the scale of hand motions by 7–20 times, increasing degrees of freedom of the microinstruments, and generating three-dimensional high-resolution images, which improve the risk of surgical tremor and protect against fatigue [9, 10].

Even if all these technical improvements enable the smallest anatomical structures to be safely operated on by experienced surgeons [11], robotics is still not established in the training of young professionals and has considerable potential to find further application in everyday surgery [12]. To date, no comparison of microsurgery with the use of a microscope and a robot by students has been described in the English literature.

The current study aimed to assess the usability and accuracy of microsurgical operation steps by students using a microscope and the Symani. In this way, new technology can be compared to established surgical techniques, helping to establish robotic systems in head and neck surgery, as well as in the education of younger surgeons, and ultimately increasing patient safety in hospitals in the long term.

## Material and methods

### Study design

**Fig. 1:**
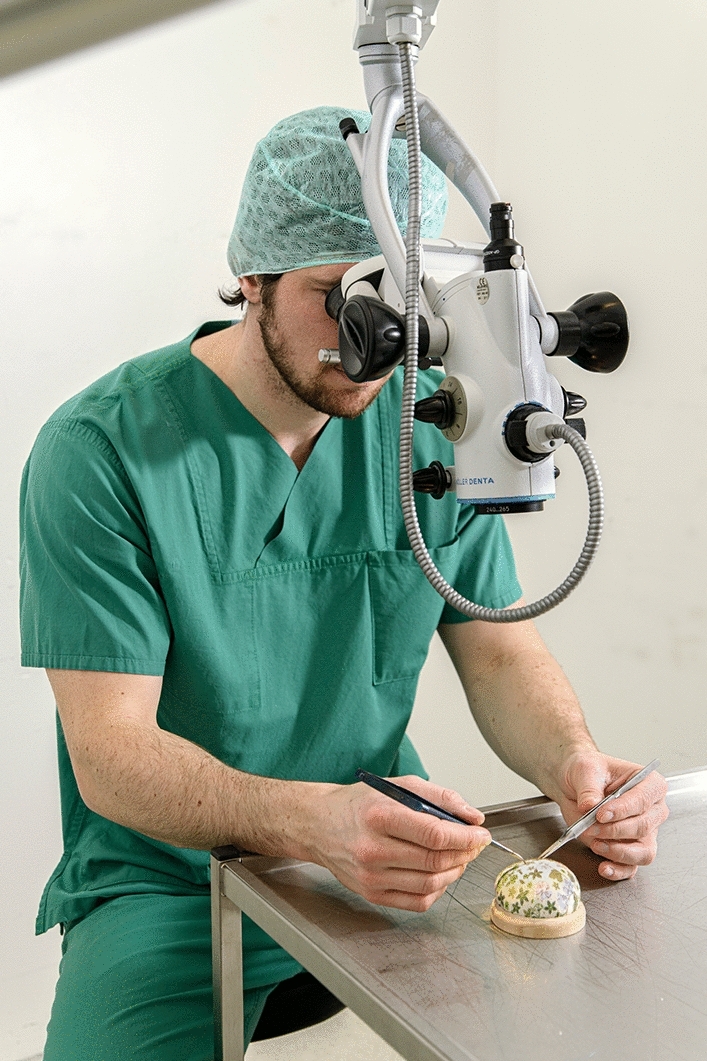
Imaged is the microscop. The student looks through the ocular with 8 × magnification and uses microneedle holder for the operation steps

**Fig. 2:**
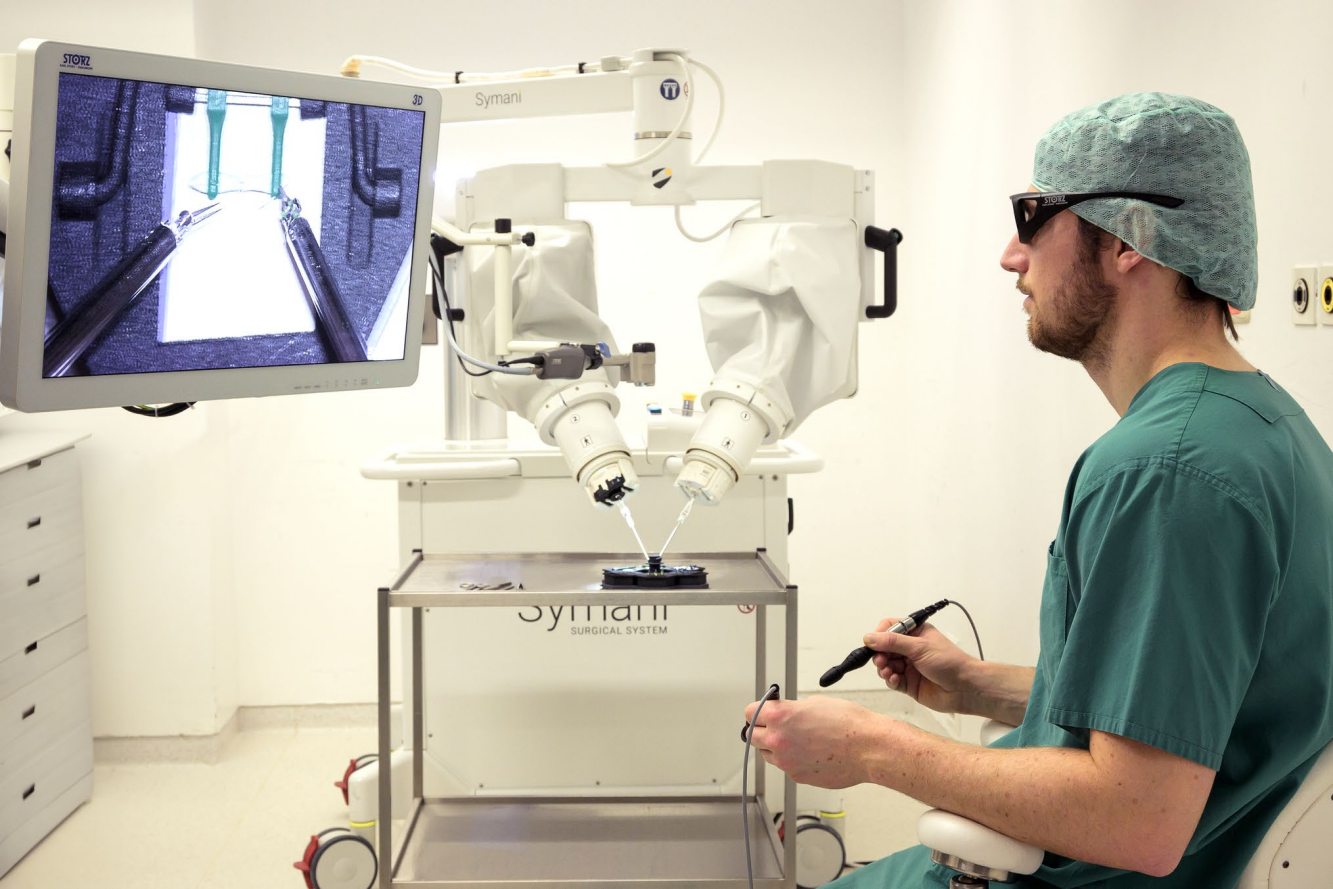
Imaged is the Symani robot. The student looks at a screen with 3D glasses and controls the 3 mm instruments using two joysticks and a foot pedal

For this study, a dental surgical operating microscope (Möller-Wedel Optical GmbH, Wedel, Germany) and the Symani from MMI were used. Three different exercises were performed to simulate the partial steps of microsurgery. For the microscope operation, the students looked through the ocular of the dental surgical operating microscope, which has an 8 × magnification, and used microneedle holders (Fig. 1). For the robotic operation, the students looked at a screen with 3D glasses and controlled the 3 mm instruments using two joysticks and a foot pedal. The used instruments were the NanoWrist Needle Holder Suture Cut and the Dilator microinstruments (Fig. 2).

Overall, 40 students (18 men and 22 women) participated in the study and operated with both systems. To avoid bias, 20 students started with the microscope and 20 with the Symani. Young professionals are familiar with the magnification of the microscope using 2.5 × magnification dental loupes in their student work. None of the students had prior experience in surgical procedures and performed their first robotic microsurgery steps. All inexperienced surgeons received individual instructions before starting the exercises. The participants did not receive any standardized pre-training; they were only given a few minutes to familiarize themselves with the system, the 3D glasses, and the joystick. As soon as the participants felt ready for the exercises, the time was started. The time of all exercises was measured and analysed. Failure criteria were not defined. The participants had unlimited time for the individual exercises. If the needle was dropped, it was retrieved with the respective surgical system.

The students were also asked how tech-savvy they considered themselves to be and whether they had any other experience in gaming, sewing, or knitting. Furthermore, the participants were asked which system they found more precise, more relaxed, faster, and easier to learn. Finally, the students were asked which system they would prefer in the clinic.

Ethical approval was obtained from the Ethics Commission of the Faculty of Medicine at the Christian-Albrechts-University, Kiel (D 408/24).

### First exercise

**Fig. 3:**
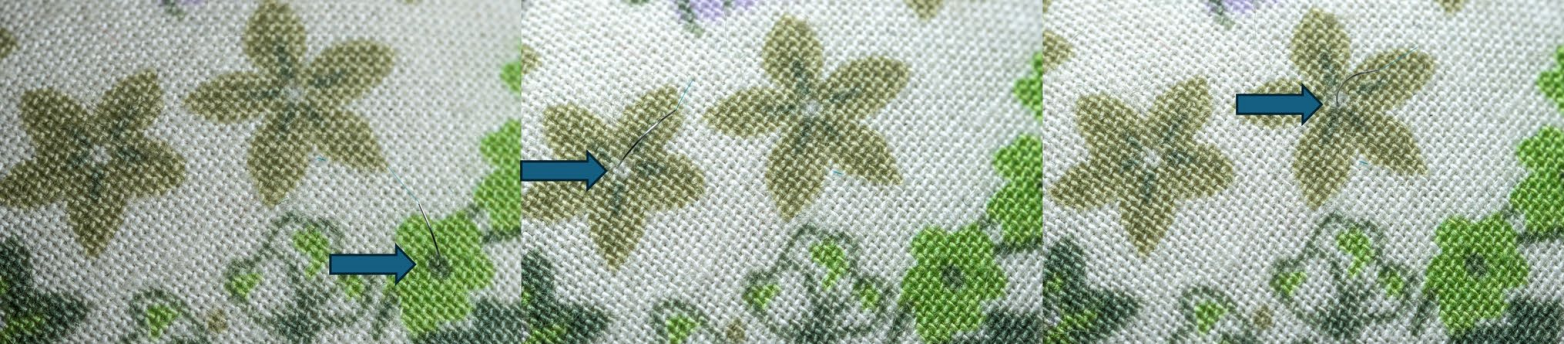
Imaged is exercise one. A 9.0 needle is grasped with the instruments and placed at the specified points (blue arrows)

A needle with a size of 9.0 had to be grasped and inserted into different patterns on a given field (Fig. 3).

### Second exercise

**Fig. 4:**
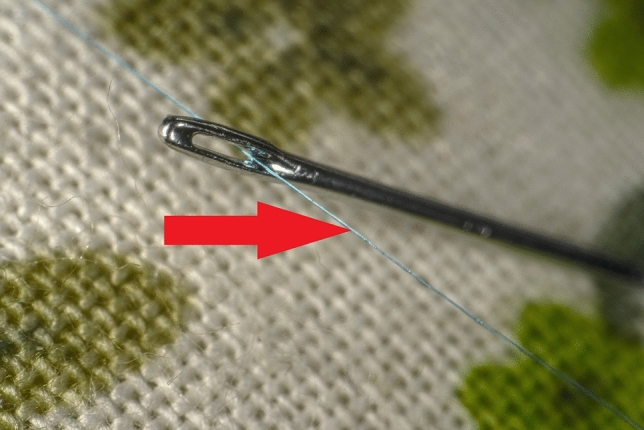
Imaged is exercise two. A thread of a 9.0 needle with a length of 5 cm (red arrow) is pulled through the eye of the needle, which is stabilized in a pincushion

A thread of a needle with a size of 9.0 and a length of five centimeters had to be grasped and passed through the eye of a needle (Fig. 4).

### Third exercise

**Fig. 5:**
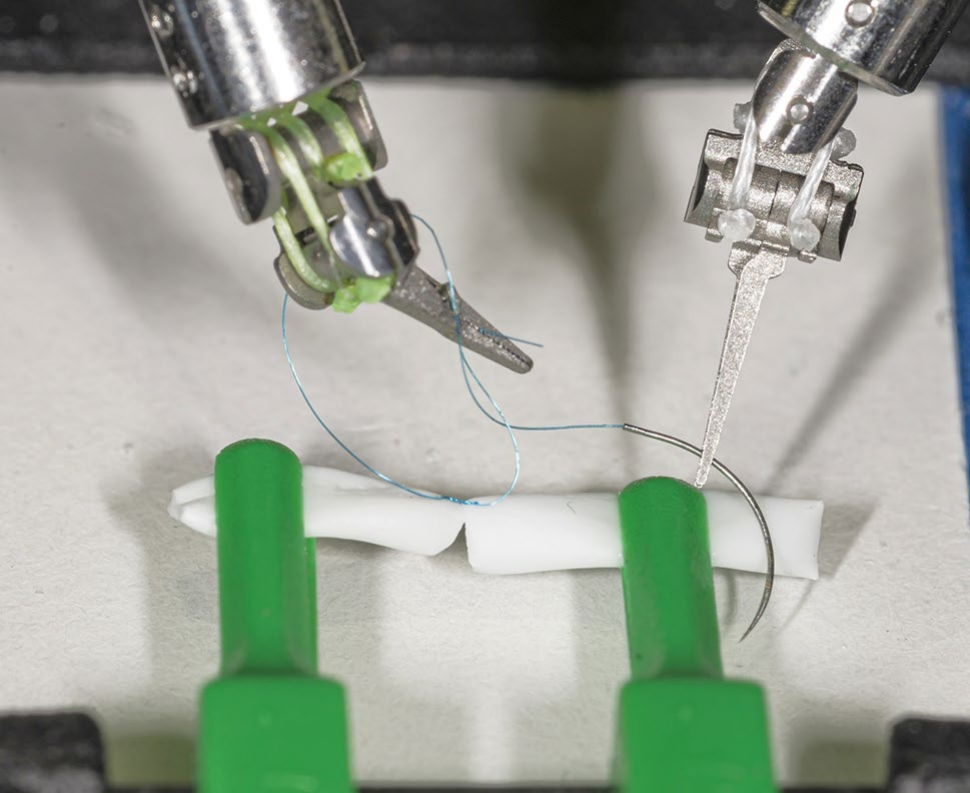
Imaged is exercise three. In the surgical simulation, an anastomosis with a diameter of 2 mm is treated. In this case, the single-button suture is performed with the 3 mm microinstruments. The ends of the vessels were adapted and held in position with a plastic approximator

A single-button suture should be used for an anastomosis. A needle with a size of 9.0 must be grasped and pierced through two vessels with a diameter of approximately 2 mm. The vessels are made of a rubber-like material that is both flexible and prone to tearing. This simulates a real vessel. The exercise was finished when the knot was tied and cut off (Fig. 5).

### Statistical analysis

Statistical analyses were performed using SPSS (IBM®, Ehningen, Germany). Normally distributed and non-normally distributed continuous variables were expressed as mean (± SD), and categorical variables were presented as total counts. The operation time of all three exercises was calculated. The relation between both groups was evaluated by sample *t*-test because there was a normal distribution (Shapiro–Wilk test). The impact of gaming, sewing, or knitting (tech-savvy group) was calculated by Mann–Whitney *U* test. Associations were considered significant when the *p*-value was < 0.05.

## Results

The young professionals were on average 24 years old (minimum 21; maximum 31). On a scale from “0” to “10”, the students gave an average of “5.4” for how tech-savvy they consider themselves to be. Regarding the other questions, 38 students (95%) found the robot more precise, and 36 (90%) found it more relaxing to operate. Only five participants (12. 5%) declared the robot to be faster in operation, and ten (25%) found it easier to learn. 31 students (77.5%) preferred the Symani as the operating system for clinical use.

**Fig. 6:**
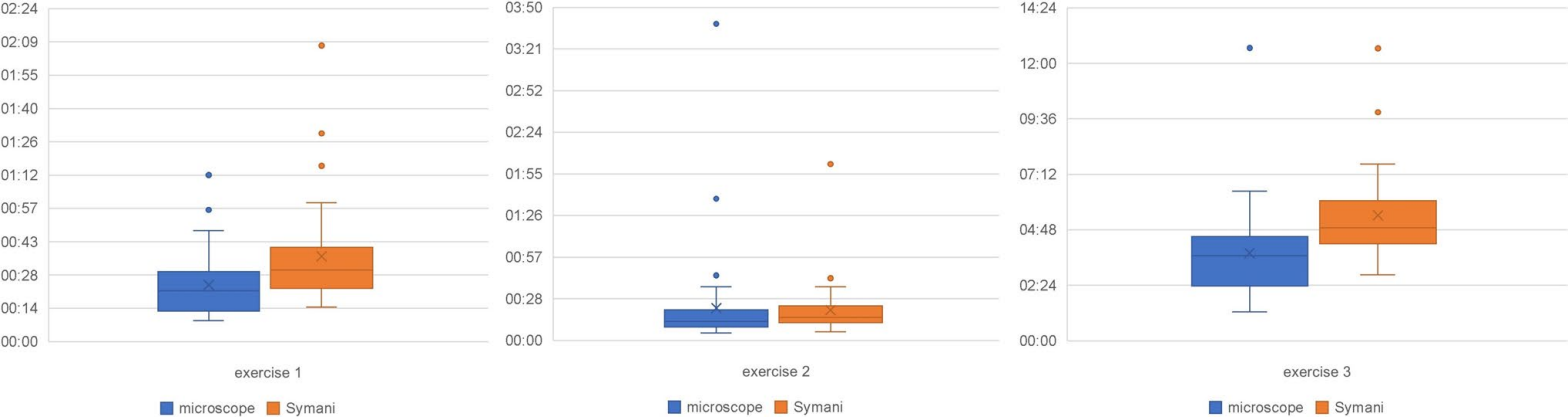
Imaged are the box plot of all measurements of the three exercises

The mean times and standard deviation for each exercise are shown in Table 1, and the box plots are shown in Fig. 6.

**Table 1:** Imaged are the mean values and the standard deviations (SD) for the operation time for the three exercises for the microscope as well as for the Symani

	Exercise 1	Exercise 2	Exercise 3
Microscope	00:24 ± 00:13 min	00:22 ± 00:35 min	03:47 ± 01:55 min
Symani	00:36 ± 00:21 min	00:21 ± 00:18 min	05:25 ± 01:59 min

### First exercise

The average exercise time was 00:24 min (minimum 00:09 min; maximum 01:12 min) for the operation step by using the microscope and 00:36 min (minimum 00:15 min; maximum 02:08 min) using the Symani. There was a significant difference in the operation time (*p* = 0.003). The average time for the tech-savvy group was significantly shorter for both the microscope and the Symani (*p* < 0.001).

### Second exercise

The average exercise time was 00:22 min (minimum 00:05 min; maximum 03:39 min) for the operation step using the microscope and 00:21 min (minimum 00:06 min; maximum 02:02 min) by using the Symani. There was no significant difference in the operation time (*p* = 0.844). There was no significant difference in the average time for the tech-savvy group for the microscope (*p* = 0.711) or the Symani (*p* = 0.493).

### Third exercise

The average exercise time was 03:47 min (minimum 01:15 min; maximum 12:40 min) for the operation step using the microscope and 05:25 min (minimum 02:51 min; maximum 12:39 min) using the Symani. There was a significant difference in the operation time (*p* < 0.001). There was no significant difference in the average time for the tech-savvy group for the microscope (*p* = 0.197) or the Symani (*p* = 0.089).

## Discussion

This study was able to show that young professionals can work effectively with a microscope as well as with a microsurgical robot, even without prior experience in microsurgery. Students can grasp and place needles and threads in a thickness of 9.0, as well as perform a single-button suture of an anastomosis in operation models. Based on the knowledge of magnifying glasses in the study, operating the microscope appears to have significant advantages in terms of operating time. These facts confirm further results of our study group. Even experienced surgeons can suture faster with a simple magnification device, such as a pair of loupes, than with a robot, but ultimately achieve similarly good surgical results [13]. Time is a good measuring tool in training to evaluate surgical skills, but there are also further objective measures of operating skills. Deepika et al. described that video recordings of robotic exercises can also help to test and improve surgical skills in neurosurgery [14]. Furthermore, Byvaltsev et al. declare that a “dry” microsurgical training model is important for training, but for more experienced surgeons, non-living tissue models as well as living models are necessary for microvascular education [15].

For this reason, we recommend extensive training on the robot to fully utilize the advantages of this new technology. Von Reibnitz et al. showed that medical students, residents, and attending physicians have a steep learning curve and can apply their robotic microsurgical skills to anastomosis using the Symani [16]. Lenfant et al. described that robotic surgery provides magnified 3D vision, improved ergonomics, and wristed instruments [17], which enable delicate manoeuvres in challenging anatomical situations and enhance surgical accuracy [18]. These could be reasons why participants prefer the robotic system for its precision and ergonomics, whereas the operation time was longer with the robot. In many medical disciplines, robotics can support precision, surgical effectiveness, and long-term patient outcomes; however, practical surgical training on the system is required before operating on patients, although implementing training situations in the clinical daily routine is very expensive [19].

In this way, robotic surgery is also gaining importance in the training of young professionals and inexperienced surgeons in other specialist areas. However, Quinn et al. showed that peg-transfer task completion by medical students was consistently faster and more accurate with a robot compared to laparoscopy and declared that these techniques should be integrated into medical education as well [20]. A steep learning curve was also described for robotic-assisted total knee arthroplasty in inexperienced surgeons; however, the complexity of a new technology must be learned [21]. This confirms our results that robotic surgery must be understood and practised but can be learned by younger surgeons immediately. Before operating on humans, detailed surgical simulations should be repeated continuously. Rabbin-Birnbaum declared that sutures on 1.5 mm anastomosis are difficult, but the learning curve for robotic microanastomoses is short and encouraging. In this way, robotics could support surgeons in the potential use of cerebrovascular bypass procedures [22]. The results of robotic surgical outcomes in several specialties, such as urology, gynaecology, and cardiac surgery, depend on various factors like surgeon experience and training cases [23]. This confirms that extensive training is essential before operations are performed and that robotic training should be integrated into the curriculum if necessary.

Students who reported gaming appeared to have advantages in operating time for simpler exercises, such as grasping and placing needles. The more complex the exercises become, the less beneficial this seems to be. Alanazi et al. reported similar results. Video gamers do not have a significant advantage in time in robotic-assisted flexible ureteroscopy [24]. Nevertheless, Cychosz et al. described how video gaming can influence arthroscopic operations, and sports requiring hand–eye coordination can also support the learning of new technologies [25]. Gupta et al. confirm this statement; for gaming and surgical procedures, similar skills, such as visuospatial abilities and hand–eye coordination, are required [26]. Türkey et al. concluded that gaming is a favorable alternative to practicing hand–eye coordination, training the understanding of 3D imaging and spatial orientation; however, the effects on surgical outcomes are highly individual and varied [27]. It is not possible to make a firm statement regarding whether these factors truly aid in microsurgery.

Moreover, all advantages of robotic microsurgery should be considered. In addition to the anastomosis of vessels, the reconstruction of nerves and supermicrosurgery on lymphatic vessels is also possible [7, 8]. The smaller and finer the anatomical reconstructions to be treated, the more advantageous the Symani system appears to be. Vessels with a diameter of less than 0.8 mm can be treated quickly and accurately with the robot compared to hand-sewn anastomosis [28]. Supermicrosurgery, in particular, is mostly limited by the precision and dexterity of the surgeon’s hands, and robot assistance can help to overcome these human limitations [29]. The participants in our study also described the robotic system as more precise and more relaxed to work with. In addition to the advantages, however, the high costs of the current systems and the mostly longer set-up time of the robotic systems must also be considered [8, 23].

Especially the precision and ergonomics in robotics microsurgery are preferred by surgeons, and often the robot system is preferred over conventional systems [30, 31]. In our study, most students were in favor of the robot and would prefer the system in everyday clinical practice. At least, most medical students are unaware of robotic surgery; however, tech-savvy young surgeons tend to have a positive attitude toward robotics [32].

The current study also has some limitations. Firstly, the operation simulation cannot be accurately compared to real life. The exercises are carried out under optimal operating conditions with no blood, no movement of the patient, reduced noise, short operation time, and little pressure to complete the operation. Secondly, this study shows only a random sample of students (*n* = 40) and a few exercises, which restricts broader applicability. Furthermore, studies of the individual subgroups and previous experiences would be helpful. For example, differences among gamers, knitters, or sewers could be investigated. Other previous professional experience or vocational training could also have an influence. A larger number of cases and complex surgical steps are necessary to confirm these surgical skills for inexperienced surgeons. In the future, a large database, multi-center studies with larger cohorts, or a prospective in vivo study for microsurgery involving both experienced and inexperienced surgeons should be established.

## Conclusions

The current study showed that students can work adequately with a microscope as well as with a microsurgical robot. Operating with a microscope seems to be an advantage due to the knowledge of using magnifying glasses during studies. However, young professionals appear to have a steep learning curve in robotic operation and can learn robotic skills rapidly. Training in robotics could be a serious alternative for learning surgical skills and inspire students to pursue careers in robotics and microsurgery. Therefore, robotic techniques should be established in students’ education. Moreover, inexperienced surgeons can train their surgical skills until they feel confident about performing their first operations on patients, and it can be objectively checked how advanced the respective surgeon is in operating with a robot.

## Data Availability

Data are provided within the manuscript or supplementary information files.
